# FGF2 Is Protective Towards Cisplatin-Induced KGN Cell Toxicity by Promoting FTO Expression and Autophagy

**DOI:** 10.3389/fendo.2022.890623

**Published:** 2022-06-16

**Authors:** Rongli Wang, Lijun Wang, Lihui Wang, Zhiwei Cui, Feiyan Cheng, Wei Wang, Xinyuan Yang

**Affiliations:** ^1^ Department of Obstetrics and Gynecology, First Affiliated Hospital, Xi’an Jiaotong University, Xi’an, China; ^2^ Department of Anesthesiology, The First Affiliated Hospital of Xi’an Jiaotong University, Xi’an, China

**Keywords:** POF, granulosa, FGF2, FTO, autophagy

## Abstract

It is widely known that chemotherapy-induced apoptosis of granulosa was the main reason for premature ovarian failure (POF). In addition, accumulating evidence has demonstrated that autophagy was involved in it. Studies before have reported that fibroblast growth factor-2 (FGF2) could attenuate cell death *via* regulating autophagy. In our previous study, FGF2 could decrease granulosa cell apoptosis in cisplatin-induced POF mice. Furthermore, obesity-associated protein [fat mass and obesity-associated protein (FTO)], which decreased significantly in POF mice, could inhibit cell apoptosis *via* activating autophagy. Moreover, downregulation of FTO could decrease the expression of paracrine factor FGF2. However, the relationship between FTO and FGF2 in granulosa cell autophagy is still unknown. In the present study, Cell Counting Kit-8 (CCK-8) and 5‐ethynyl‐2‐deoxyuridine (EdU) assays showed that exogenous addition of FGF2 could promote cisplatin-induced injured granulosa cell proliferation. Western blotting indicated that FGF2 could inhibit apoptosis of injured granulosa cells *via* autophagy. Inhibition of autophagy by chemicals suppressed the effect of FGF2 and promoted injured cell apoptosis. In addition, the expression of FTO was decreased in injured cells, and FGF2 addition could reverse it. Overexpression of FTO reduced injured cell apoptosis *via* activating the autophagy process. Our findings indicated that FGF2 activates autophagy by regulating the expression of FTO, thereby reducing the apoptosis of the injured cells.

## Introduction

With the incidence rate of gynecological malignant tumors increasing and the younger age of onset, chemotherapy-induced premature ovarian failure (POF) is becoming more prevalent (1). POF is a disease that leads to infertility, osteoporosis, cardiovascular disease, and cognitive impairment (2, 3). Studies before have indicated that chemotherapy-induced POF has a close relationship with the apoptosis and injury of granulosa (1, 4). In addition, autophagy, which is mainly induced in granulosa, is closely related to the induction of apoptosis (5, 6).

Autophagy is a conserved catabolic process that plays a vital role in the regulation of cellular homeostasis. The effect of autophagy could be either destructive or protective for the host (7). In general, cells deliver the aggregated proteins and damaged organelles into lysosomes to generate metabolites *via* autophagy (8, 9). In response to chemotherapy, autophagy is also a double-edged sword (10). Studies before have demonstrated that the promotion of autophagy could prevent cisplatin-induced cytotoxicity in normal cells (11). While in cancer cells, inhibition of autophagy could decrease cisplatin efficiency (12). In the ovaries, autophagy is involved in the selection of dominant follicles. In addition, autophagy mainly occurs in granulosa cells and directly participates in follicular atresia (13). In the process of autophagy, the expression of microtubule-associated protein 1 light chain 3 (LC3-II) increased, which means the formation of a double-membrane autophagosome vesicle. Moreover, the increased Beclin1 indicates the initiation of macroautophagy. The p62/SQSTM1 presented the autophagy completed, and the autophagy flux was not impaired (14).

Fibroblast growth factor-2 (FGF2), which is also named basic fibroblast growth factor (bFGF), is a member of FGFs. FGFs could modulate ovarian function, such as proliferation, differentiation, and granulosa apoptosis (15). Previous studies have shown that FGF2 secreted from the stem cells could repair ovarian injury and restore ovarian function in POF animals (16, 17). In bovine ovarian granulosa cells, miR-21-3p decreased the expression of FGF2, suppressed autophagy, and inhibited cell proliferation (18). In SH-SY5Y cells, overexpression of FGF2 plays a protective role in Parkinson’s disease by inhibiting apoptosis and promoting autophagy (19). Whether the exogenous addition of FGF2 could reduce chemotherapy-induced granulosa cell apoptosis and improve ovarian function *via* autophagy is still unknown.

RNA methylation on the sixth Natom of adenylate (m^6^A) was one of the most abundant modifications of RNA (20). FTO (fat mass and obesity-associated protein), a vital member of m^6^A, could catalyze the demethylation of m^6^A and causes multiple malformations and growth retardation (21), especially in reproduction. For example, in male germ cells, downregulation of FTO suppresses spermatogonial proliferation (22). In female oocytes, abnormal FTO was related to oocyte maturation disorder (23). Furthermore, the expression of FTO decreased significantly in the ovarian tissue of POF patients and mouse models (23). A previous study has reported that FTO could participate in the autophagy process in many diseases (24, 25). Downregulation of FTO could promote cisplatin-induced cytotoxicity (26) and decrease the stem cell expression of paracrine factor FGF2 (27). We wonder whether FTO is involved in cisplatin-induced granulosa cell damage through autophagy. The relationship between FGF2 and FTO in cisplatin-induced injured granulosa cells also needs to be explored.

The study aimed to unveil the mechanism of FGF2 in restoring the function of cisplatin-induced injured granulosa cells. First, we identified that FGF2 could inhibit injured cell apoptosis *via* promoting autophagy. Then, we found that FTO may be a downstream target of FGF2 and further explored the effect of FTO in granulosa cell autophagy.

## Materials and Methods

### Cell Culture and Reagents

Human ovarian granulosa cell line KGN (Procell CL-0603) was obtained from Procell Life Science & Technology Co., Ltd. (Wuhan, China), and cultured in Dulbecco's modified eagle medium (DMEM)/F12 (Hyclone, Logan, UT) that was supplemented with 10% fetal bovine serum (FBS; Sijiqing, China) at 5% CO_2_ in 37°C. The medium was changed every 48 h.

We purchased FGF2 from Origene (TP750002S) and dissolved it in an aqueous buffer [0.1% bovine serum albumin (BSA)]. The concentration of Cis-diamminedichloroplatinum-II. (CDDP) (Sigma–Aldrich, St. Louis, MO, USA) used in this study was 10 µmol L^-1^ in saline. Plasmid for overexpression of FTO and its corresponding control vector were obtained from Miaoling (Wuhan, China). Small interfering RNAs (siRNAs) for FTO or FGF2 and non-specific negative control siRNA (si-NC) were synthesized by Ribobio (Guangzhou, China). The transfection reagent used in this study was Lipofectamine 2000 (Thermo Fisher Scientific, Waltham, MA, USA; 11668019). The inhibitor of autophagy 3-metyladenine (3-MA) was obtained from MCE, while chloroquine (CQ) was obtained from Sigma. Rapamycin, an activator of autophagy, was purchased from MCE.

### Cell Proliferation Assay

Ten thousand cells/per well were plated in a 96-well plate for the cell proliferation assay, and cells were cultured at 5% CO_2_ and 37°C. According to the manufacturer’s instructions, cell viability was determined by the Cell Counting Kit-8 (CCK-8; Dojindo, Kumamoto, Japan). All experiments were conducted in triplicate.

### Plasmid Transfection and RNA Interference

According to the manufacturer’s instruction, the granulosa cells (2 × 10^5^ cells/well) were seeded in a 6-well plate, and when cells reached 60%–80% confluence, they were transfected with 50 nmol L^-1^ siRNA using Lipofectamine 2000. For the plasmids, cells were transfected with 4 µg plasmids by 3 µl Lipofectamine 2000 per well. After transfection for 6 h, the transfection mixture was removed, and the cells were incubated with a complete medium for another 24 h.

### Western Blotting

Cells were harvested, and the radioimmunoprecipitation (RIPA) lysis buffer was added, which added the protease inhibitor cocktail (1:50) and phenylmethanesulfonyl fluoride (PMSF) (1:100) in advance. The concentration of the total proteins was measured using the Bicinchoninic Acid Assay (BCA) kit (Beyotime, Nanjing, China). A total of 30 µg proteins per sample were subjected to the sodium dodecyl sulfate-polyacrylamide gel electrophoresis (SDS-PAGE). Then, proteins were separated by gel electrophoresis and transferred to the polyvinylidene difluoride (PVDF) membrane. The membranes were blocked with 5% non-fat skim milk in TBST for 90 min at room temperature and incubated at 4°C overnight with the corresponding primary antibody. The primary antibodies used in this study were as follows: BAX (1:1,000, 50599-2-Ig, Proteintech, China); Bcl-2 (1:1,000, 12789-1-AP, Proteintech, China); LC3 (1:1,000, 3868, CST, USA); Beclin1 (1:1,000, ab210498, Abcam, USA); p62 (1:1,000, 23214, CST, USA); FTO (1:1,000, ab126605, Abcam, USA); FGF2 (1:1,000, ab208687, Abcam, USA); β-actin (1:1,000, 66099-1-Ig, Proteintech, China). Finally, the membranes were washed with Tris Buffered Saline (TBS) with Tween (20 mmol/L Tris (pH 7.4), 150 mmol/L sodium chloride, 0.1% Tween 20) three times, incubated with a secondary antibody (1:5,000, SA00001-2, Proteintech, China) at room temperature for 1 h, and visualized using an enhanced chemiluminescence method (Thermo, MA, USA).

### RNA Isolation, Reverse Transcription, and Quantitative Real-Time PCR

Total RNA from cultured cells was extracted using TRIzol reagent (Invitrogen, Co., Carlsbad, CA, USA), and the purity and concentration were detected by the NanoDrop 2000 spectrophotometer (Nanodrop Technologies, Wilmington). Here, 500 ng RNA was reverse transcribed according to the manufacturer’s instruction (Takara). The PCR was carried out using an SYBR Premix Ex Taq™ on a StepOne Plus Real-time PCR system (Life Technologies, Carlsbad, CA, USA). The primers used in this study were as follows: BAX (forward, 5′–AGTGGCAGCTGACATGTTTT–3′; reverse, 5′–GGAGGAAGTCCAATGTCCAG–3′); Bcl-2 (forward, 5′–TTCCACGCCGAAGGACAGCG–3′; reverse, 5′–GGCACTTGTGGCGGCCTGA–3′); FTO (forward, 5′–CTTCACCAAGGAGACTGCTATTTC–3′; reverse, 5′–CAAGGTTCCTGTTGAGCACTCTG–3′); FGF2 (forward, 5′–AGAAGAGCGACCCTCACATCA–3′; reverse, 5′–CGGTTAGCACACACTCCTTTG–3′); GAPDH (forward, 5′–AAAATCAAGTGGGGCGATGCT–3′; reverse, 5′–TGGTTCACACCCATGACGAAC–3′).

The sequences of siFGF2 are as follows:

siRNA sequence 1: 5′–GAAGGAAGATGGAAGATTA–3′;

siRNA sequence 2: 5′–TGGGCAGTATAAACTTGGA–3′;

siRNA sequence 3: 5′–GCTACAACTTCAAGCAGAA–3′.

### 5‐Ethynyl‐2‐Deoxyuridine Staining

The 5‐ethynyl‐2‐deoxyuridine (EdU) staining was conducted using an EdU Apollo567 kit (Ribobio Co., Ltd.). After treatment, the cells were incubated with EdU (1:1,000, 50 μmol/L) for 2 h, washed twice with phosphate buffer saline (PBS), followed by fixing with 4% paraformaldehyde for 30 min at room temperature. The cells were washed with glycine (2 mg/ml) for 5 min and then permeabilized with 0.5% Triton X-100 in PBS for 10 min and incubated in Apollo^®^ reaction cocktail (100 μl) for 30 min at room temperature while being protected from light. Then, cells were again washed twice with PBS. Subsequently, cells were incubated with Hoechst 33342 for 30 min for DNA staining. Finally, the cells were washed with PBS 3 times, and images were captured using fluorescent microscopy (Olympus, Japan).

### Cell Apoptosis Analysis Using Flow Cytometry

Cell apoptosis was detected using an Annexin V‐PE/7‐AAD Apoptosis Detection Kit (BD Biosciences, Shanghai, China). Before transfection or treatment, the cells were cultured in a 6-well plate overnight. When they reached 60%–80% confluence, cells were treated with cisplatin/FGF2 or transfection with plasmids/siRNA for 48 h. Then, the cells were harvested and centrifuged at 1,000g for 5 min. The cells were washed with ice-cold PBS twice, and the cells were resuspended with 1× binding buffer. Then, 400 µl of the cells were incubated with 5 µl PE‐Annexin V and 5 μl 7‐aminoactinomycin D (7‐AAD) for 15 min at room temperature in the dark. Finally, 100 µl of 1× binding buffer was added to each tube, and the FAC-Scan system (Becton Dickinson, Mountain View, CA, USA) was used to analyze cell apoptosis.

### Immunofluorescence

Cells were cultured on cover glasses in a 6-well plate and then washed with PBS 3 times. The cells were fixed in 4% paraformaldehyde for 30 min at room temperature and permeabilized with 0.5% Triton X-100 in PBS for 20 min. Then, the cells were blocked with 5% BSA for 60 min at room temperature and incubated with rabbit anti-LC3A/B antibody at 4°C overnight. On the second day, washed the cells twice with PBS and incubated them with anti-rabbit Alexa Fluo488 (Invitrogen) for 60 min at a room temperature in the dark. Finally, the cells were mounted on the cover glasses with 4',6-diamidino-2-phenylindole (DAPI) (Invitrogen), and the image was obtained on a fluorescence microscope (Olympus Inc., USA). The number of LC3 puncta was qualified manually.

### Monodansylcadaverine Staining

We used monodansylcadaverine (MDC) staining for the detection of autophagic vesicles in autophagic cells. MDC could be used as a tracer of autophagic vesicles. All acidic vacuoles in the cells were visualized by MDC staining. According to the manufacturer’s instructions, 2 × 10^5^ cells were plated in a 6-well plate overnight before group treatments. After different treatments, cells were stained with MDC (Beyotime, C3018S, Nanjing, China) for 30 min at 37°C in the dark, washed with assay buffer three times, and visualized using a fluorescence microscope (Olympus Inc., USA).

### Statistical Analysis

Every experiment was carried out in triplicate. Statistical analysis was done with GraphPad Prism 5. The Student’s t-test was used to compare the difference between the two experimental groups. One-way ANOVA test was applied to compare multiple groups. The data were represented as mean ± SEM, and P-value <0.05 was considered statistically significant.

## Results

### Screening the Concentration of Fibroblast Growth Factor-2

**Figure 1:**
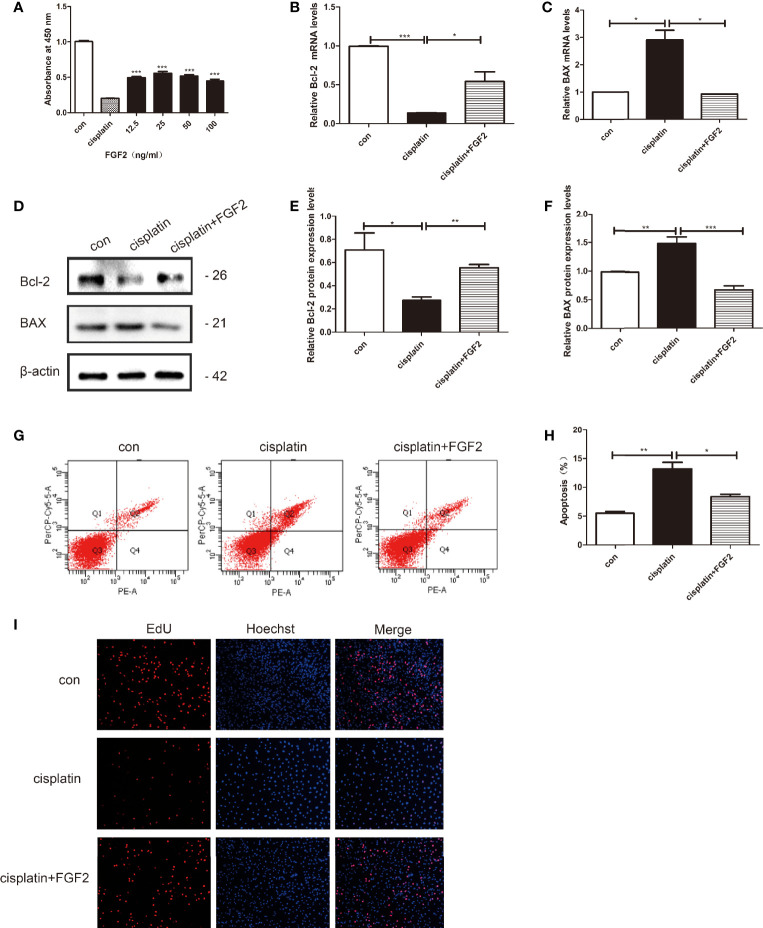
FGF2 promotes cisplatin-induced injured granulosa cell proliferation and inhibits its apoptosis. Cisplatin-induced injured granulosa cell was generated by treating KGN cells with 10 μM cisplatin for 24 h. **(A)** CCK-8 assay was performed to measure the rate of injured KGN cell proliferation adopted with different concentrations of FGF2. The results indicated that treatment with 25 ng/ml FGF2 significantly increased cell viability. **(B, C)** The mRNA expression levels of Bcl-2 and BAX were assessed using qRT-PCR and normalized to GAPDH. **(D–F)** The protein expressions of Bcl-2 and BAX were determined by Western blotting with β-actin as a loading control. **(G, H)** Flow cytometry assays were performed to determine the effect of FGF2 on injured KGN cell apoptosis. A quantitative analysis of the rate of apoptosis was performed. **(I)** EdU labeling assay was used to detect cell viability. Hoechst was used as a DNA/nuclear stain. One-way ANOVA followed by Tukey’s multiple comparison test. The results were shown as mean ± SEM from 3 independent experiments (α = 0.05, *P < 0.05, **P < 0.01, ***P < 0.001). FGF2, Fibroblast Growth Factor-2; CCK-8, Cell Counting Kit-8; qRT-PCR, Quantitative Real-Time PCR; EdU, 5‐Ethynyl‐2‐Deoxyuridine Staining.

To screen the optimum concentration of FGF2, KGN cells were treated with cisplatin (10 µM) for 24 h, and then cells were treated with different concentrations of FGF2 (0, 12.5, 25, 50, 100 ng/ml) for 72 h (28). The CCK-8 assay was applied to test the recovery of cell viability. The results indicated that injured cell treatment with 25 ng/ml FGF2 significantly increased the viability of the cells (**Figure 1A**). Then, we choose the 25 ng/ml FGF2 as an effective concentration in the later experiments.

### Fibroblast Growth Factor-2 Promoted Injured Cell Proliferation and Inhibited Its Apoptosis *In Vitro*

KGN cells were divided into three groups: control group (nontreated), cisplatin-treated group (10 µM), and cisplatin plus FGF2 (25 ng/ml) group. qRT-PCR and Western blotting results indicated that compared to the control group, both the mRNA and protein expression levels of BAX were increased in the cisplatin-treated group, and the expression levels of Bcl-2 were decreased (**Figures 1B–F**). However, when the injured cells were treated with FGF2 (25 ng/ml), the expression levels of BAX and Bcl-2 could reverse to the control levels (**Figures 1B–F**). Flow cytometry analysis and EdU staining were also used to test the effect of FGF2 on cisplatin-induced injured granulosa cells. Flow cytometry analysis indicated that compared to the cisplatin-treated group, FGF2 treatment decreased cell apoptosis (**Figures 1G, H**). EdU staining showed that cisplatin treatment inhibited cell proliferation, while FGF2 treatment could promote injured cell proliferation (**Figure 1I**). These results indicated that FGF2 plays a protective role in injured KGN cells.

### The Effect of Fibroblast Growth Factor-2 on Injured Cell Autophagy

**Figure 2:**
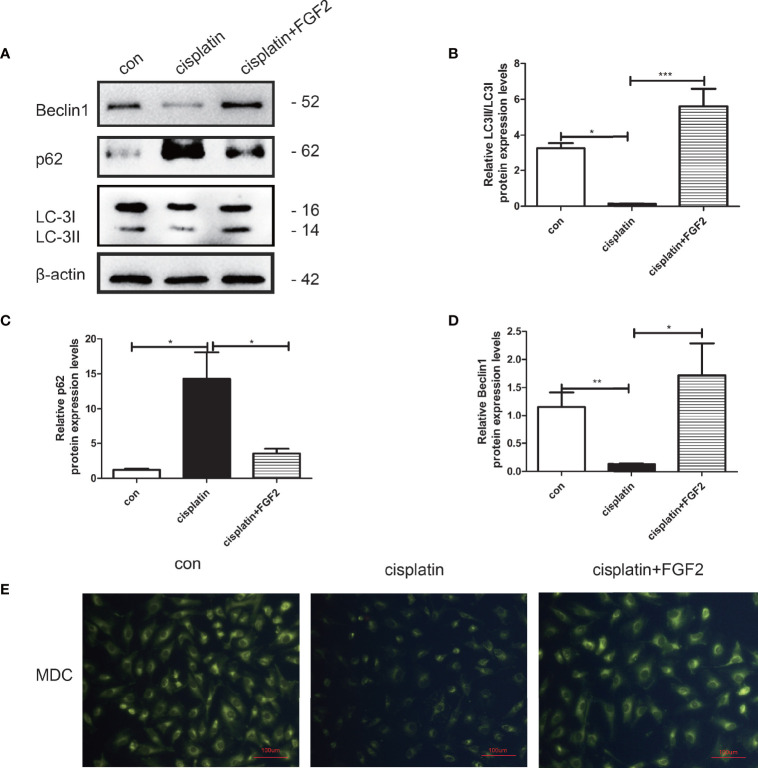
FGF2 promoted autophagy in injured KGN cells. **(A–D)** Western blot assay was used to assay LC3, Beclin1, and p62 expression. The results indicated that compared to the control groups, the autophagy was decreased in cisplatin-induced injured KGN cells. When injured KGN cells were treated with FGF2, the autophagy increased. **(E)** Representative images of KGN cells were treated with cisplatin and cisplatin plus FGF2, and then cells were stained with MDC (scale bar: 100 μm). One-way ANOVA followed by Tukey’s multiple comparison test. The results were shown as mean ± SEM from 3 independent experiments (α = 0.05, *P < 0.05, **P < 0.01, ***P < 0.001).

To explore the effect of FGF2 on injured cell autophagy, KGN cells were divided into three groups: 1) control group (nontreated); 2) cisplatin-treated group (10 µM); 3) cisplatin plus FGF2 (25 ng/ml) group. Western blotting indicated that FGF2 promotes the expression of LC3B and beclin1 and negatively regulates p62 expression compared to cisplatin-treated only (**Figures 2A–D**). Furthermore, MDC staining was performed to observe acidic vesicular organelles (AVOs), which indicates the level of autophagy. As shown in **Figure 2E**, compared to the control group, KGN cells treated with cisplatin showed less accumulation of autophagic vacuoles. However, after cisplatin treatment, FGF2 addition could reverse it. In summary, these findings suggested that FGF2 increased injured KGN cell autophagy levels.

### Inhibition of Autophagy Attenuates Fibroblast Growth Factor-2-Induced Antiapoptosis Effects in Injured Granulosa Cells

**Figure 3:**
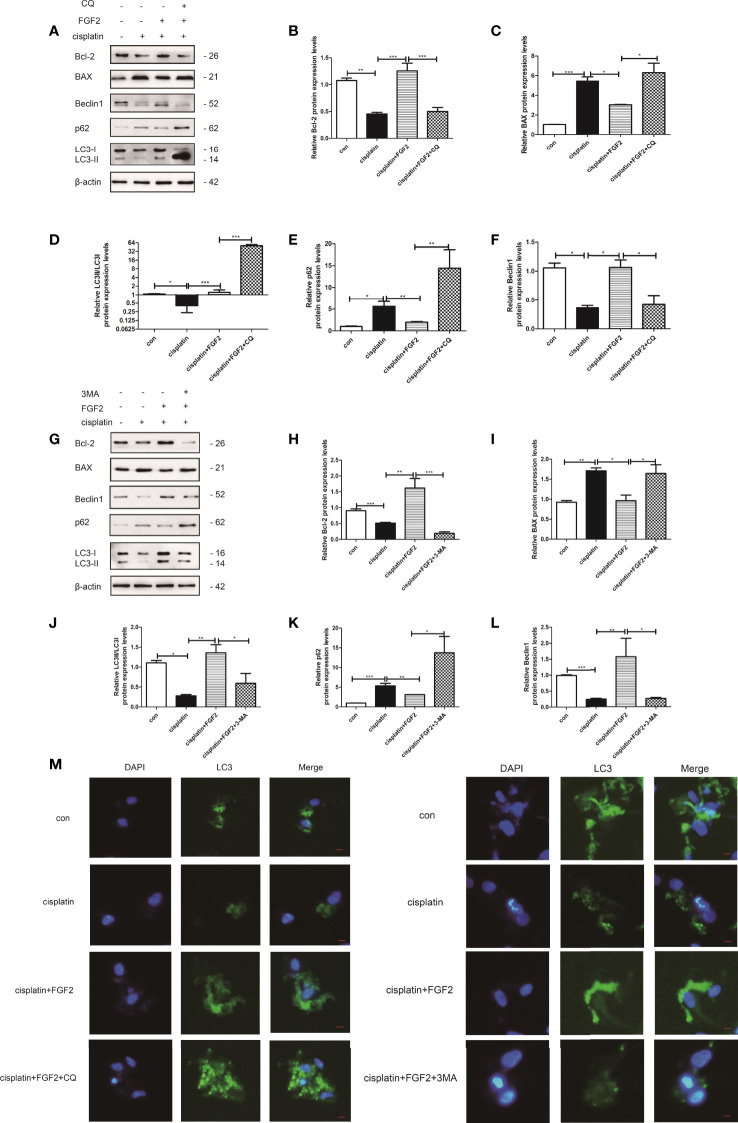
Inhibition of autophagy by 3-MA and CQ attenuated the protective effects of FGF2 on injured KGN cells. **(A)** The injured KGN cells were treated with FGF2 (25 ng/ml) for 72 h and incubated with 3-MA (10 mM) for another 24 h. The expression of Bcl-2, BAX, LC-3, Beclin1, and p62 was monitored by Western blot. Here, β-actin was used as a loading control. **(B–F)** Quantitative comparison of the different expression levels of Bcl-2, BAX, LC3, Beclin1, and p62. One-way ANOVA followed by Tukey’s multiple comparison test. The results were shown as mean ± SEM from three independent experiments (α = 0.05, *P < 0.05, **P < 0.01, ***P < 0.001). **(G)** The injured KGN cells were treated with FGF2 (25 ng/ml) for 72 h and incubated with CQ (50 μM) for another 24 h. The expression of Bcl-2, BAX, LC-3, Beclin1, and p62 was monitored by Western blot. Here, β-actin was used as a loading control. **(H–L)** Quantitative comparison of the different expression levels of Bcl-2, BAX, LC3, Beclin1, and p62. One-way ANOVA followed by Tukey’s multiple comparison test. The results were shown as mean ± SEM from three independent experiments (α = 0.05, *P < 0.05, **P < 0.01, ***P < 0.001). **(M)** Representative immunofluorescence image of GFP-LC3 puncta in KGN cells with different treatments (scale bar: 20 μm).

KGN cells were divided into 1) control group (nontreated); 2) cisplatin-treated group (10 µM); 3) cisplatin plus FGF2 (25 ng/ml) group; 4) cisplatin plus FGF2 and then plus 3-MA (5 mM) or CQ (100 µM) group. Our results demonstrated that both 3-MA and CQ inhibited autophagy in the FGF2-treated group, accompanied by the upregulation of apoptosis protein BAX and the downregulation of antiapoptosis protein Bcl-2 (**Figures 3A–L**). Furthermore, immunofluorescence was applied to detect the formation of GFP‐LC3 punctate. Fluorescence microscopy showed that the level of GFP‐LC3 punctate formation was lower in the cisplatin-treated group than that in the control group. However, when FGF2 was added to the injured KGN cells, the level of GFP‐LC3 punctate formation was increased. When the cells were further treated with 3-MA, the GFP‐LC3 punctate decreased again. And when cells were further treated with CQ, the GFP‐LC3 punctate increased significantly (**Figure 3M**). These results demonstrated that inhibiting the FGF2-mediated autophagy decreased the protective role of FGF2 on injured KGN cells.

### Downregulation of Fibroblast Growth Factor-2 in Injured Cells Promoted Injured Cell Apoptosis *via* Attenuating Autophagy

**Figure 4:**
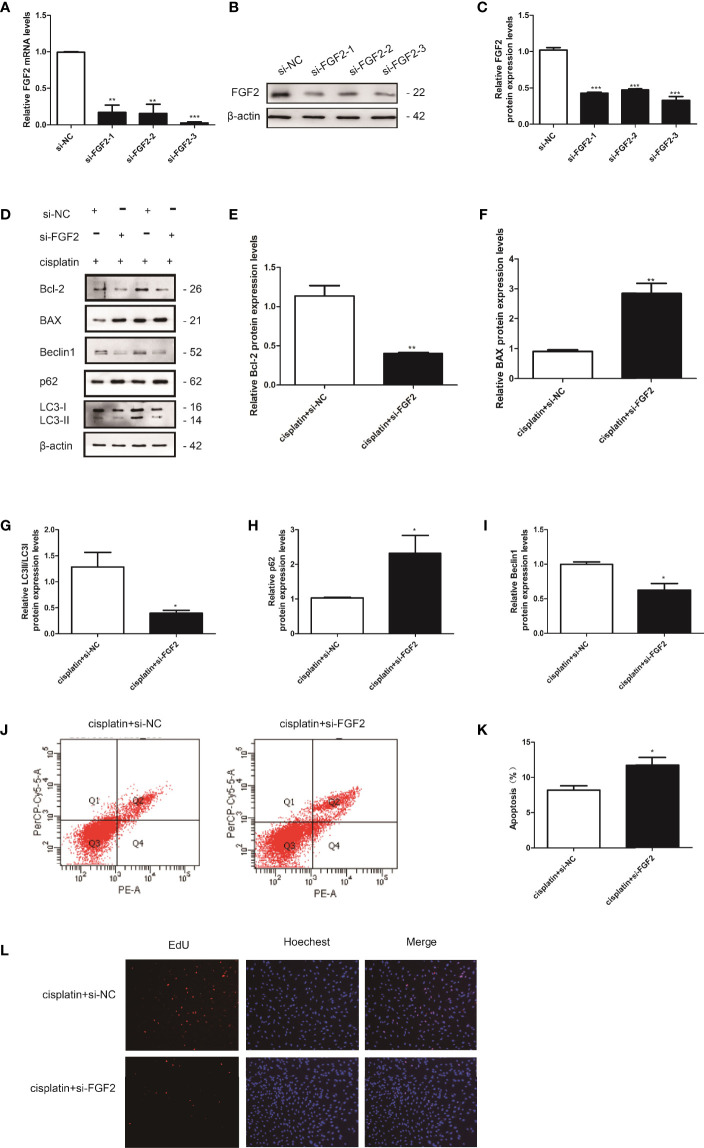
Knockdown of FGF2 in injured KGN cells promoted cell apoptosis *via* inhibiting autophagy. **(A–C)** The transfection efficiency was confirmed by qRT-PCR and Western blotting. **(D)** The injured KGN cells were transfected with si-FGF2 (50 nM) for 48 h. The expression of Bcl-2, BAX, LC-3, Beclin1, and p62 was monitored by Western blot. Here, β-actin was used as a loading control. **(E–I)** Quantitative comparison of the different expression levels of Bcl-2, BAX, LC3, Beclin1, and p62. One-way ANOVA followed by Tukey’s multiple comparison test. The results were shown as mean ± SEM from three independent experiments (α = 0.05, *P < 0.05, **P < 0.01, ***P < 0.001). **(J, K)** Flow cytometry assays were performed to determine the effect of si-FGF2 on injured KGN cell apoptosis. A quantitative analysis of the rate of apoptosis was performed. **(L)** EdU labeling assay was used to detect cell viability. Hoechst was used as a DNA/nuclear stain.

We also examined the effect of downregulation of FGF2 on injured KGN cell autophagy, and the si-FGF2 was applied. The transfected efficiency was determined by qRT-PCR and Western blotting (**Figures 4A–C**). Then, the cells were divided into three groups: 1) control group (nontreated); 2) cisplatin-treated group (10 µM); 3) cisplatin plus si-FGF2 (50 nM) group. The results showed that compared to the cisplatin-treated group, downregulation of FGF2 in injured KGN cells declined the expression of LC3B and beclin1 and promoted the expression of p62, accompanied by decreased Bcl-2 expression and increased expression of BAX (**Figures 4D–I**). Flow cytometry analysis and EdU staining were also used to test the effect of si-FGF2 on cisplatin-induced injured KGN cells. Flow cytometry analysis indicated that compared to the cisplatin-treated group, si-FGF2 treatment promoted cell apoptosis (**Figures 4J, K**). EdU staining showed that si-FGF2 cotreatment with cisplatin could further inhibit injured KGN cell proliferation (**Figure 4L**). These results indicated that downregulation of FGF2 in injured KGN cells promoted cell apoptosis *via* inhibiting autophagy.

### Activation of Autophagy by Rapamycin Attenuated the Proapoptotic Effects of Small Interfering RNA-Fibroblast Growth Factor 2 (si-FGF2) on Injured KGN Cells

**Figure 5:**
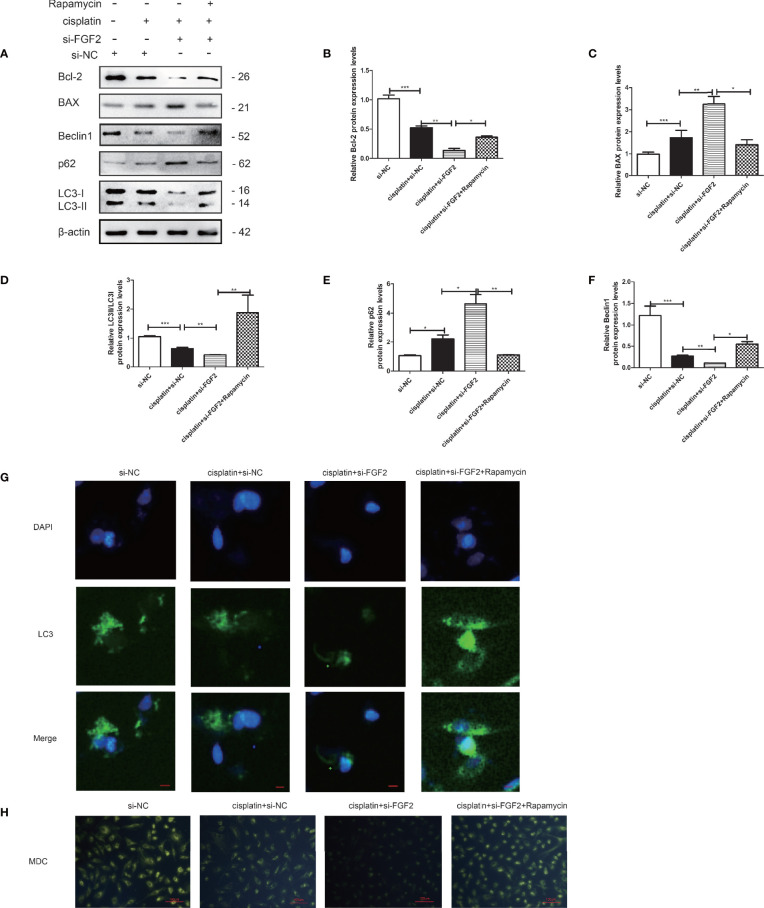
Activation of autophagy by rapamycin attenuated the proapoptotic effects of si-FGF2 on injured KGN cells. After the injured KGN cells were transfected with si-FGF2 for 48 h, rapamycin was added and cultured for another 4–6 h. **(A)** The expression of Bcl-2, BAX, LC-3, Beclin1, and p62 was monitored by Western blot. Here, β-actin was used as a loading control. **(B–F)** Quantitative comparison of the different expression levels of Bcl-2, BAX, LC3, Beclin1, and p62. One-way ANOVA followed by Tukey’s multiple comparison test. The results were shown as mean ± SEM from three independent experiments (α = 0.05, *P < 0.05, **P < 0.01, ***P < 0.001). **(G)** Representative immunofluorescence image of GFP-LC3 puncta in KGN cells with different treatments (scale bar: 20 μm). **(H)** Representative images of KGN cells were treated with si-NC, si-NC plus cisplatin, cisplatin plus si-FGF2, and cisplatin plus si-FGF2 and rapamycin, and then cells were stained with MDC (scale bar: 100 μm).

The cells were divided into four groups: 1) control group (nontreated); 2) cisplatin-treated group (10 µM); 3) cisplatin plus si-FGF2 (50 nM) group; 4) cisplatin plus si-FGF2 (50 nM) and then plus rapamycin (100 nM). The results demonstrated that by activating the autophagy in the si-FGF2-treated group using rapamycin, the expression levels of BAX decreased and the expression of Bcl-2 increased (**Figures 5A–F**). Fluorescence microscopy showed that the level of GFP‐LC3 punctate formation was lower in the cisplatin-treated group than that in the control group. When the injured KGN cells were transfected with si-FGF2, the level of GFP‐LC3 punctate further decreased. When the cells were further treated with rapamycin, the GFP‐LC3 punctate increased significantly (**Figure 5G**). Furthermore, MDC staining was performed to observe AVOs. As shown in **Figure 5H**, compared to the si-NC group, KGN cells treated with cisplatin showed less accumulation of autophagic vacuoles. In the group of cisplatin plus si-FGF2, the accumulation of autophagic vacuoles further declined. When the cells were further treated with rapamycin, the autophagic vacuoles increased significantly.

### Fibroblast Growth Factor-2 Facilitates the Expression of FTO in Injured Granulosa Cells

**Figure 6:**
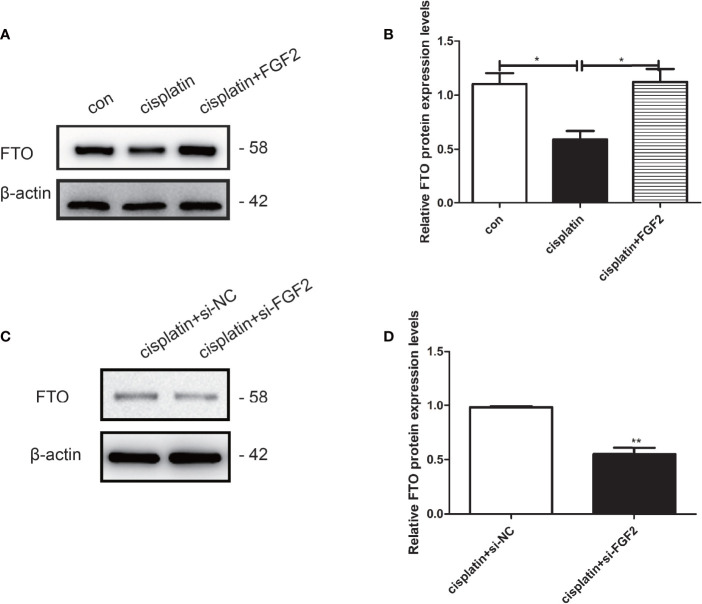
FGF2 facilitated the expression of FTO in injured KGN cells. **(A, B)** Western blot indicated that exogenous addition of FGF2 into injured KGN cells resulted in upregulation of FTO than those of injured cells. One-way ANOVA followed by Tukey’s multiple comparison test. The results were shown as mean ± SEM from three independent experiments (α = 0.05, *P < 0.05). **(C, D)** Western blot indicated that transient transfection of si-FGF2 resulted in downregulation of FTO in injured KGN cells when compared to those transiently transfected with si-NC. Student’s t-test. The results were shown as mean ± SEM from three independent experiments (α = 0.05, **P < 0.01).

To explore the effects of FGF2 on FTO expression in injured KGN cells, FGF2 was added to the cisplatin-treated KGN cells. The results indicated that FGF2 could promote the expression of FTO in injured KGN cells compared to those treated with cisplatin only (**Figures 6A, B**). However, downregulation of FGF2 in injured KGN cells decreased the expression of FTO (**Figures 6C, D**).

### The Effects of FTO on Granulosa Cell Autophagy

**Figure 7:**
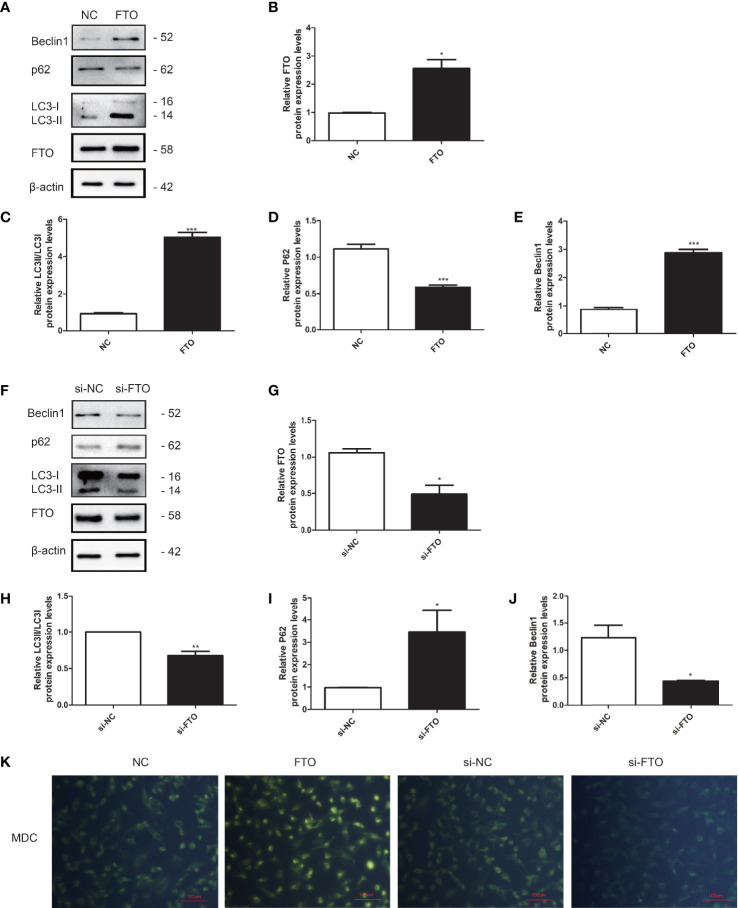
FTO promoted autophagy in KGN cells. **(A, B)** The transfection efficiency of FTO was measured by Western blot. **(C–E)** Quantitative comparison of the different expression levels of LC3, Beclin1, and p62. Student’s t-test. The results were shown as mean ± SEM from three independent experiments (α = 0.05, *P < 0.05, ***P < 0.001). **(F, G)** The transfection efficiency of si-FTO was measured by Western blot. **(H–J)** Quantitative comparison of the different expression levels of LC3, Beclin1, and p62. Student’s t-test. The results were shown as mean ± SEM from three independent experiments (α = 0.05, *P < 0.05, **P < 0.01). **(K)** Representative images of KGN cells were treated with NC, FTO, si-NC, and si-FTO, and then cells were stained with MDC (scale bar: 100 μm).

We further investigated the role of FTO on KGN cell autophagy. The transfection efficiency of FTO plasmid and si-FTO and the negative controls were confirmed by qRT-PCR and Western blotting (**Figures 7A, B, F, G**). The results indicated that overexpression of FTO could promote the LC3 and Beclin1 expression and decrease the p62 expression (**Figures 7C–E**). Downregulation of FTO got the opposite results (**Figures 7H–J**). MDC staining showed that FTO could promote the accumulation of autophagic vacuoles compared to the NC group. Downregulation of FTO decreased the accumulation of autophagic vacuoles (**Figure 7K**).

### Overexpression of FTO Could Restore Cisplatin-Induced Apoptosis *via* Promoting Autophagy

**Figure 8:**
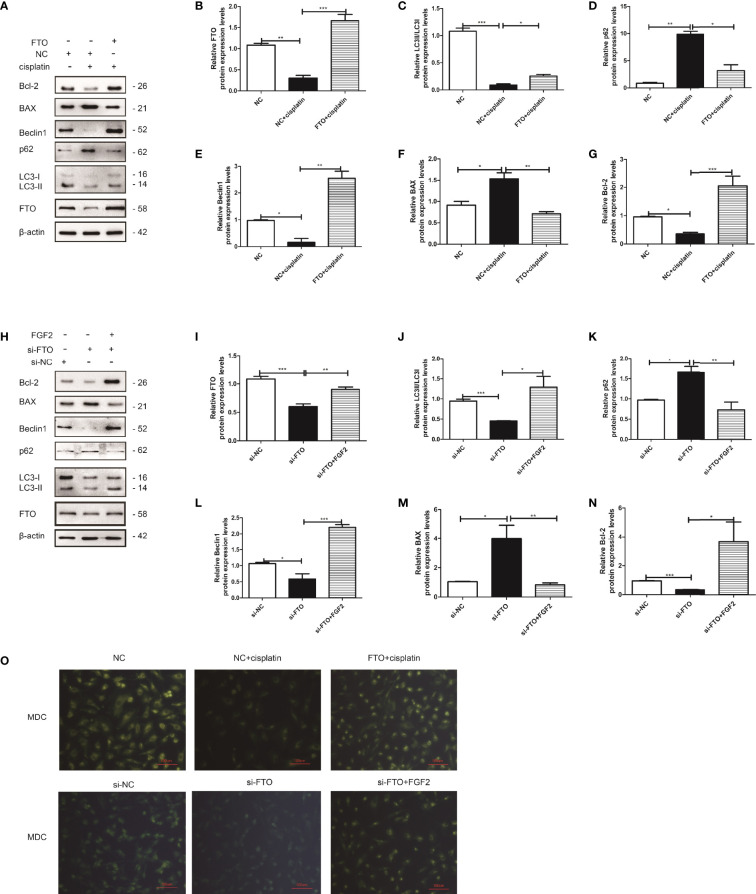
FTO reversed injured KGN cell autophagy and decreased cell apoptosis. **(A)** Western blot and gain- and loss-of-function studies were conducted to explore the role of FTO in injured KGN cells. **(B–G)** Quantitative comparison of the different expression levels of FTO, Bcl-2, BAX, LC3, Beclin1, and p62. One-way ANOVA followed by Tukey’s multiple comparison test. The results were shown as mean ± SEM from three independent experiments (α = 0.05, *P < 0.05, **P < 0.01, ***P < 0.001). **(H)** Western blot and gain- and loss-of-function studies were conducted to show that FGF2 promoted injured KGN cell autophagy by regulating FTO expression. **(I–N)** Quantitative comparison of the different expression levels of FTO, Bcl-2, BAX, LC3, Beclin1, and p62. One-way ANOVA followed by Tukey’s multiple comparison test. The results were shown as mean ± SEM from three independent experiments (α = 0.05, *P < 0.05, **P < 0.01, ***P < 0.001). **(O)** Representative images of KGN cells were treated with NC, NC plus cisplatin, and FTO plus cisplatin or si-NC, si-FTO, and si-FTO plus FGF2, and then cells were stained with MDC (scale bar: 100 μm).

We downregulated the expression of FTO in pharmacology or gene levels. FTO was restored *via* FTO plasmid or FGF2. KGN cells were divided into three groups: NC group, NC plus cisplatin group, and FTO plasmid group (**Figure 8A**) or si-NC group, si-FTO group, and si-FTO plus FGF2 group (**Figure 8H**). We found that the downregulation of FTO could decrease cell autophagy and promote cell apoptosis by increasing the expression of BAX and inhibiting the expression of Bcl-2. While restoring the expression of FTO could reverse it, which promoted cell autophagy and decreased cell apoptosis (**Figures 8A–N**). MDC staining showed the same results (**Figure 8O**).

## Discussion

FGF2 is a pleiotropic cytokine that plays a vital role in repairing and regenerating damaged tissues (29, 30), including POF (17). Our observations suggest that FGF2 has a protective role in cisplatin-induced granulosa cell apoptosis. This is consistent with our previous study that FGF2 had a protective effect on ovarian dysfunction in C57BL/6 mice administered cisplatin 2 mg/kg for 7 consecutive days (17). However, the mechanisms of FGF2 in recovering cisplatin-induced cell/tissue injury are still unknown.

Granulosa cells, which lie outside the oocyte, could synthesize and release a series of growth factors, hormones, and nutrition to regulate oocyte development (31, 32). Previous studies have demonstrated that the pathogenesis of POF was the acceleration of follicle atresia (33, 34). While the apoptosis of granulosa cells may be the main cause of follicle atresia (4). Injured granulosa cell could be an *in vitro* model of POF (1). Maintaining the homeostasis of autophagy is a necessary condition for the ovary to function. Numerous studies have demonstrated the cross-link between autophagy and follicle development and atresia (35). Autophagy is mainly induced in ovarian granulosa cells (36–38). Precise regulation of autophagy in granulosa cells plays an important role in the growth and differentiation of oocytes (6). In addition, autophagy is also closely related to granulosa cell apoptosis, which is involved in preserving the primordial follicle pool of young rats and eliminating inferior follicles during development (39). Previous studies have shown that the abnormal regulation of autophagy in granulosa cells is involved in the occurrence and development of POF (40). When the autophagy level of granulosa cells continues to increase, the programmed death program is activated, which reduces the expression of Bcl-2 and induces the apoptosis of granulosa cells, leading to follicular atresia and POF (41).

FGF2, as a novel autophagy activator, has been reported in several studies. In SH-SY5Y cells, FGF2 could promote autophagy, inhibit apoptosis, and finally exert positive effects on Parkinson’s disease (PD) (19). Furthermore, FGF2 has been reported to be a regulator of p62 (42, 43). Consistent with them, our study demonstrated that the exogenous addition of FGF2 protects granulosa cells from cisplatin-induced toxicity to inhibit apoptosis and promote cell survival by promoting autophagy. Compared to the cisplatin-treated groups, the expression level of BAX decreased in the exogenous addition of FGF2 groups, and the expression level of Bcl-2 increased, accompanied by an increased expression level of LC3-II and inhibition of p62. When an inhibitor of autophagy was added to the injured cells simultaneously, the apoptosis of the cells increased. However, when the autophagy inhibitors CQ and 3-MA were used, there was a different effect on the expression of LC3-II. The reason is that 3-MA is a pan-Phosphatidylinositol 3-kinase (PI3K) inhibitor, which inhibits autophagy at the early stage of autophagic flux *via* blocking autophagosome formation and participates in the initiation of autophagosome formation (44–46). While the CQ treatment blocks the fusion of autophagosomes with lysosomes and subsequent lysosomal protein degradation, thereby promoting autophagosome accumulation (44, 47, 48). Downregulation of FGF2 in injured granulosa cells obtained the opposite results, which promoted injured cell apoptosis *via* inhibiting autophagy. The activator of autophagy rapamycin could reverse the effect of si-FGF2 on injured cells.

Furthermore, our previous studies have demonstrated that FGF2 secreted from menstrual-derived stem cells (MenSCs) reduced granulosa cell apoptosis in POF mice (17). In addition, MenSCs promote the recovery of chemotherapy-induced POF mice ovarian and cisplatin-induced injured granulosa cells *via* regulating the expression of FTO (49). In this study, we found that FGF2 increases FTO expression in injured granulosa cells. By downregulating FGF2 in injured granulosa cells, the protein expression level of FTO decreased. Overexpression of FTO could activate autophagy and decrease granulosa cell apoptosis. While downregulation of FTO inhibited granulosa cell proliferation and promoted cell apoptosis *via* inhibiting autophagy. Exogenous addition of FGF2 could reverse the inhibitory effect of FTO downregulation on autophagy and then decreasing cell apoptosis and promoting their proliferation. These results indicated that FGF2 may promote the proliferation of cisplatin-induced injured granulosa cells and decrease their apoptosis by regulating the expression of FTO and activating autophagy. In summary, the FGF2/FTO axis plays a protective role in cisplatin-induced injury in granulosa cells *via* activating autophagy.

## Data Availability Statement

The original contributions presented in the study are included in the article/supplementary materials, further inquiries can be directed to the corresponding author/s.

## Author Contributions

XY and WW conceived the study. RW, LJW, and LHW performed the experiment. RW, ZC, and FC collected and analyzed the data. RW and WW wrote the article. All authors contributed to the article and approved the submitted version.

## Funding

This study was supported by the National Natural Science Foundation of China (No. 81571393), Shaanxi Provincial Natural Science Foundation (No. 2019KW-065), and Project A of the First Affiliated Hospital of Xi’an Jiaotong University (XJTU-2021-02).

## Conflict of Interest

The authors declare that the research was conducted in the absence of any commercial or financial relationships that could be construed as a potential conflict of interest.

## Publisher’s Note

All claims expressed in this article are solely those of the authors and do not necessarily represent those of their affiliated organizations, or those of the publisher, the editors and the reviewers. Any product that may be evaluated in this article, or claim that may be made by its manufacturer, is not guaranteed or endorsed by the publisher.
